# Salivary MicroRNA Reflects Neurodevelopment and Oral Health Traits in Children With Autism

**DOI:** 10.1016/j.identj.2026.109632

**Published:** 2026-05-20

**Authors:** Omar Omar, Reem Yussuf AlJindan, Sumit Rajinder, Balu Kamaraj, Jehan AlHumaid

**Affiliations:** a Department of Biomedical Dental Sciences, College of Dentistry, Imam Abdulrahman Bin Faisal University, Dammam, Saudi Arabia; b Department of Microbiology, College of Medicine, Imam Abdulrahman Bin Faisal University, Dammam, Saudi Arabia; c Department of Preventive Dental Sciences, College of Dentistry, Imam Abdulrahman Bin Faisal University, Dammam, Saudi Arabia; d Department of Dental Education, College of Dentistry, Imam Abdulrahman Bin Faisal University, Dammam, Saudi Arabia

**Keywords:** MicroRNAs, Autism spectrum disorder, Biomarkers, RNA sequencing, Periodontal diseases, Dental caries

## Abstract

**Introduction and aims:**

Autism spectrum disorder (ASD) is a multifactorial neurodevelopmental condition associated with poor oral health in children. ASD lacks sensitive, non-invasive diagnostic tools, and the relationship between neurodevelopment and oral health remains unclear. This observational study aimed to identify salivary microRNAs (miRNAs) differentially expressed between children with ASD and healthy controls and to determine whether these profiles are associated with dental caries and periodontal status.

**Methods:**

Unstimulated saliva was collected from 26 children with ASD and 20 healthy controls and subjected to small RNA sequencing. Differentially expressed miRNAs were identified and functionally annotated. Oral health was assessed using dmft/DMFT and periodontal indices, and associations between miRNA expression patterns and clinical parameters were evaluated.

**Results:**

A total of 125 salivary miRNAs were differentially expressed between ASD and control groups (FDR < 0.05). Principal component analysis showed clear group separation, with PC1 accounting for 46.5% of total variance. Several dysregulated miRNAs were linked to neurodevelopmental and immune regulatory pathways and overlapped with neurological and inflammatory conditions. After miRDB/SFARI filtering, 40 ASD-relevant miRNAs were prioritized, including 21 upregulated and 19 downregulated candidates; representative examples were hsa-miR-145-5p (log2FC 2.11, adjusted *P* = 1.74E-08) and hsa-miR-200a-5p (log2FC -0.58, adjusted *P* = 6.11E-06). Children with ASD had higher dmft/DMFT scores and poorer periodontal/behavioural outcomes, with dmft/DMFT differing significantly between groups (*P* = .02). In exploratory pooled-cohort regression analysis, miR-151a-3p and behaviour remained significant predictors of dmft/DMFT (adjusted R^2^ = 0.31, *P* = .003).

**Conclusions:**

Children with ASD exhibit a distinct salivary miRNA profile with increased caries burden and gingival inflammation, indicating shared molecular influences on neurodevelopment and oral disease susceptibility.

**Clinical Relevance:**

Salivary miRNAs offer a non-invasive biomarker platform to support earlier ASD identification, individualized preventive dental strategies, and interdisciplinary management of this high-risk paediatric population.

## Introduction

Autism Spectrum Disorder (ASD) is a complex neurodevelopmental disorder characterized by deficits in social interaction, communication, and restricted or repetitive behaviours. Globally, ASD affects approximately 1 in 100 children.^1^ Within Saudi Arabia, recent studies have reported a prevalence of around 2.5% among children, underscoring its regional clinical significance.^2^

Early diagnosis of ASD is crucial, as it permits timely intervention and support that can substantially improve developmental outcomes.^3^^,^^4^ However, many children experience delays in diagnosis, often not diagnosed until after 3 years of age. These delays not only postpone intervention but also lead to increased healthcare utilization and subsequent cost burdens, as well as exacerbation of mental health and social difficulties.^5^

Given these challenges, there is an urgent need for early, objective, and non-invasive biomarkers of ASD. While genetic studies have identified numerous risk genes, their heterogeneity limits their diagnostic utility.^6^^,^^7^ Attention has turned to epigenetic regulators, such as microRNAs (miRNAs), small non-coding RNAs that modulate post-transcriptional gene expression and are essential for brain development, neuronal plasticity, and immune signaling.^8^^,^^9^ Importantly, miRNAs are detectable in readily accessible peripheral biofluids such as saliva, enabling non-invasive sampling, a critical advantage for paediatric populations and individuals with sensory sensitivities commonly observed in ASD.^10^^,^^11^ Recent studies have demonstrated altered salivary miRNA profiles in children with ASD, suggesting their promise as potential diagnostic tools.^10^^,^^12^

Furthermore, while ASD patients may not exhibit distinct dental anomalies, they are considered a high-risk group for caries and periodontal disease due to sensory, behavioural, and dietary factors.^13^^,^^14^ Emerging evidence links oral health with neurodevelopmental and immune pathways, both of which are regulated by miRNAs.^15^ Yet no detailed study has comprehensively examined the interrelationships among salivary miRNA expression, ASD pathology, and oral health status.

Here, we aimed to identify salivary miRNAs differentially expressed in children with ASD compared to controls and assess their relevance to ASD-related genes and neurodevelopmental pathways. Additionally, we aimed to explore the correlation between miRNA expression and oral health indices (dmft/DMFT and periodontal scores) and the potential of salivary miRNAs as dual biomarkers for ASD and its oral comorbidities. By bridging neuroscience, epigenetics, and dentistry, this study introduces a novel framework to understand the biological convergence between neurodevelopmental risk and oral disease susceptibility in ASD.

## Materials and methods

This human observational study conforms to the STROBE guidelines. The study was conducted in accordance with the principles of the Declaration of Helsinki and approved by the Institutional Review Board (IRB) at the University (IRB-2021-02-463). Written informed consent was obtained from the children’s legal guardians.

### Study participants and clinical assessment

A total of 46 children were enrolled, comprising 26 individuals diagnosed with autism spectrum disorder (ASD) and 20 neurotypical controls. The mean age was 11.5 ± 3.01 years in the ASD group (range 5-16 years) and 8.9 ± 3.2 years in the control group (range 4-16 years). The ASD cohort included 20 males and 6 females, while the control cohort included 10 males and 10 females. As this was an exploratory observational study, the final sample size was determined by the number of eligible participants recruited during the study period after informed consent and was consistent with sample sizes reported in previous similar salivary miRNA studies in children with ASD.^16^

ASD diagnoses were established based on DSM-5 criteria using the Autism Diagnostic Observation Schedule (ADOS) and the Childhood Autism Rating Scale (CARS), administered by trained clinicians. Exclusion criteria included syndromic autism, history of major systemic illness, or ongoing infections. Neurodevelopmental functioning was evaluated using the Vineland Adaptive Behaviour Scales, through structured parent interviews, assessing communication, socialization, and daily living skills.

To document the child’s response to the oral examination, 2 complementary chairside measures were recorded: (1) temper classification (5 categories: friendly, fearful, shy, active, or resistant/uncooperative), and (2) behavioural cooperation using the Frankl Behaviour Rating Scale (4 categories: definitely negative, negative, positive, or definitely positive).^17^^,^^18^

### Oral health examination

Two independent examiners conducted oral examinations. Dental caries were recorded using the dmft/DMFT indices following the World Health Organization (WHO) guidelines. Periodontal health was assessed using a modified Periodontal Disease Index (PDI). Inter-examiner reliability for behaviour, dmft/DMFT, and periodontal scoring was assessed by blinded re-scoring of 10 cases by 3 independent examiners.

### Saliva collection and RNA isolation

Unstimulated saliva samples were collected using sterile flocked swabs rotated under the tongue for ∼30 seconds, avoiding contact with the tongue and teeth. Samples were immediately stabilized in RNA-preserving solution (DNA/RNA Shield; Zymo Research) and stored at −80°C until processing. Total RNA, including small RNAs, was extracted using the SPLIT RNA Extraction Kit (Lexogen GmbH) following the manufacturer’s protocol. RNA concentration and purity were assessed using NanoDrop 2000c spectrophotometry (Thermo Fisher), while RNA integrity was confirmed via Fragment Analyser (Agilent Technologies) using the DNF-472 RNA HS Kit according to the manufacturer’s instructions.

### Small RNA library preparation and sequencing

Library preparation was performed using the Lexogen Small RNA-Seq Library Prep Kit optimized for biofluid samples. Residual genomic DNA was removed via DNase I treatment. The resulting cDNA libraries were size-selected and validated using a Fragment Analyser with the DNF-474 HS-DNA kit, ensuring insert size distribution and purity. Sequencing was conducted on an Illumina NextSeq 2000 platform with a 75-nucleotide single-end read configuration. An average depth of 8 to 10 million reads per sample was achieved.

### Bioinformatics and functional enrichment analysis

Raw sequencing reads were quality-filtered and adapter-trimmed using Cutadapt.^19^ Reads were aligned to the miRBase v22human mature miRNA reference^20^ using Bowtie.^21^ Expression levels were normalized and analysed for differential expression using the DESeq2 package^22^ in R. A Benjamini-Hochberg false discovery rate (FDR) correction was applied, and miRNAs with adjusted *P*-values <.05 were considered statistically significant.

Target genes of differentially expressed miRNAs were predicted using miRDB^23^ and cross-referenced with SFARI Gene,^24^ a curated database of autism risk genes. Functional annotation was performed using GeneCodis4 webtool,^25^ and enrichment analyses were conducted for Gene Ontology (GO) categories (biological process, molecular function, and cellular component) and pathway databases, including KEGG, Reactome, and WikiPathways. Disease and human phenotype ontology enrichments were evaluated using RNAdisease, Online Mendelian Inheritance in Man (OMIM), and Human Phenotype Ontology (HPO) databases.

### Salivary microbial profiling

An additional aliquot of the saliva was processed for microbial analysis using the Bruker MALDI Biotyper (Matrix-Assisted Laser Desorption Ionization-Time of Flight Mass Spectrometry, MALDI-TOF MS). The core study cohort comprised 46 children (26 ASD and 20 controls). For salivary microbial profiling, an additional 5 ASD samples were available; therefore, bacterial detection analyses were performed on 31 ASD samples and 20 control samples. These additional ASD samples were included only in the microbiological analysis and were not part of the miRNA sequencing, clinical correlation, or regression analyses. Bacterial identification across samples was achieved by matching mass spectra against an established microbial database, providing genus- and species-level resolution. Identification scores ≥ 2.0 were considered reliable for species-level identification.

### Statistical analysis

All statistical analyses were performed using IBM SPSS Statistics (v30) and GraphPad Prism (v11). Inter-examiner reliability analyses were performed in SPSS (3 blinded examiners; 10 cases) using Kendall’s W for ordinal scores (behavioural cooperation and periodontal) and ICC (2-way random, absolute agreement; single measures) for dmft/DMFT. Agreement was excellent for behaviour (W = 0.93, *p* = .003) and periodontal score (W = 0.99, *p* = .002), and good for dmft/DMFT (ICC = 0.86; 95% CI: 0.63-0.96; *p* < .001). Between-group comparisons were performed using Fisher’s exact test for categorical variables (behavioural cooperation, gingival index, and periodontal index) and an unpaired t-test with Welch’s correction for dmft/DMFT. Spearman’s rank correlation coefficients were calculated for the 6 predefined correlation families corresponding to Figure 5A–F. To account for multiple testing, *p*-values were adjusted within each correlation family using the Benjamini–Hochberg false discovery rate (FDR) method. Both nominal *p*-values and FDR-adjusted q-values are reported in the Supplementary Information (Appendix Raw Data File 7). miRNA variables demonstrating significant associations with dmft/DMFT scores, together with other clinically relevant variables, were further evaluated using multiple linear regression models as potential predictors of dmft/DMFT scores. Because these models were used as follow-up exploratory analyses on a reduced candidate set rather than as a separate high-dimensional screening step, no additional multiplicity correction was applied to the regression coefficients. Nominal statistical significance was set at *p* < .05 (2-tailed), while FDR-adjusted significance thresholds were applied where specified.

## Results

**Fig. 1:**
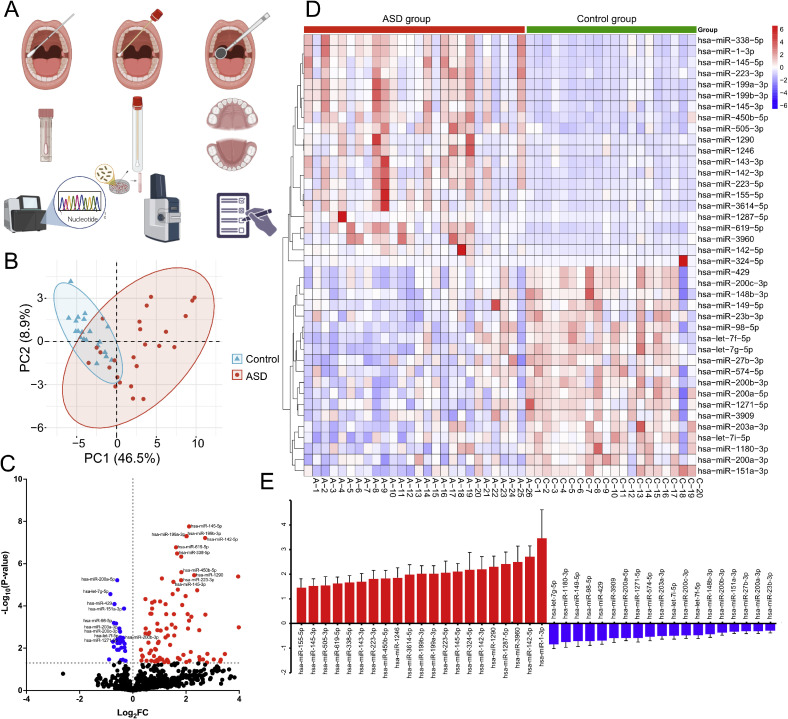
**Experimental design and salivary microRNA profiling in ASD and control children.** (A) Overview of study workflow. Saliva was collected from children diagnosed with autism spectrum disorder (ASD) and age-matched healthy controls. Samples were processed for small RNA sequencing, salivary microbial identification using MALDI-TOF MS, and clinical evaluation of oral health. (B) Principal component analysis (PCA) based on normalized miRNA expression levels shows separation between ASD (n = 26) and control (n = 20) samples. (C) Volcano plot depicting log2 fold change versus –log10 FDR-adjusted *p*-values for all detected miRNAs. Red and blue dots represent significantly up- and down-regulated miRNAs, respectively (FDR < 0.05). (D) Heatmap of significantly dysregulated miRNAs (FDR < 0.05), clustered across samples. (E) Bar graph of top differentially expressed miRNAs (mean log2 fold change ± SEM). Parts of the schematic were created using BioRender.com.

A schematic representation of the conducted study design and methodological workflow is illustrated in Figure 1A.

### Salivary miRNA profiles robustly differentiate ASD from healthy controls

Small RNA sequencing of saliva samples from ASD (n = 26) and control (n = 20) children identified 125 significantly dysregulated miRNAs (FDR < 0.05; Appendix Raw Data File 1). These included 97 upregulated and 28 downregulated miRNAs in ASD. Principal component analysis (PCA) based on normalized miRNA expression levels revealed clear group separation, with PC1 accounting for 46.5% of total variance, underscoring the discriminative potential of salivary miRNAs (Figure 1B). Differential expression was visualized via a volcano plot and hierarchical clustering heatmaps (Figures 1C and D), highlighting a distinct miRNA expression signature in ASD.

Using miRDB target prediction and cross-referencing with the SFARI database of autism-associated genes, we identified that 40 of the 125 dysregulated salivary miRNAs in ASD children were prioritized as ASD-relevant candidates (Table 1, Figure 1E; Appendix Raw Data File 1). Of these, 21 were upregulated (Figure 1E, Supplementary Figure S1) and 19 were downregulated (Figure 1E, Supplementary Figure S2) in ASD compared to controls. Notably, this subset includes several top dysregulated miRNAs, such as miR-155-5p, miR-142-3p, and miR-223-5p (upregulated), as well as miR-151a-3p, miR-1271-5p, and miR-149-5p (downregulated). These miRNAs are functionally linked to immune modulation, synaptic signaling, and mucosal integrity, underscoring their potential involvement in the neurodevelopmental and systemic features of ASD.

**Table 1:** Key salivary miRNAs differentially expressed in ASD and prioritized through miRDB/SFARI filtering are shown with their corresponding log2 fold change (log2FC) and Benjamini–Hochberg false discovery rate (FDR)-adjusted *p*-values

Up-regulated miRNA	log2FC	Adjusted *p*-value	Down-regulated miRNA	log2FC	Adjusted *p*-value
hsa-miR-145-5p	2.11	1.74E-08	hsa-miR-200a-5p	-0.58	6.11E-06
hsa-miR-199a-3p	2.02	5.02E-08	hsa-let-7g-5p	-0.84	2.74E-05
hsa-miR-199b-3p	2.02	5.00E-08	hsa-miR-429	-0.69	8.15E-05
hsa-miR-142-5p	2.72	6.14E-08	hsa-miR-151a-3p	-0.33	1.34E-04
hsa-miR-619-5p	1.62	1.69E-07	hsa-miR-98-5p	-0.71	6.34E-04
hsa-miR-338-5p	1.66	3.29E-07	hsa-miR-203a-3p	-0.5	1.17E-03
hsa-miR-450b-5p	1.82	2.54E-06	hsa-miR-200c-3p	-0.48	1.65E-03
hsa-miR-1290	2.31	3.51E-06	hsa-let-7f-5p	-0.47	3.07E-03
hsa-miR-223-3p	1.82	6.11E-06	hsa-miR-200b-3p	-0.37	3.25E-03
hsa-miR-145-3p	1.53	7.28E-06	hsa-miR-1271-5p	-0.58	3.88E-03
hsa-miR-143-3p	1.69	1.69E-05	hsa-miR-1180-3p	-0.75	4.91E-03
hsa-miR-1287-5p	2.42	1.81E-05	hsa-let-7i-5p	-0.49	5.72E-03
hsa-miR-3614-5p	1.98	3.43E-05	hsa-miR-200a-3p	-0.31	6.18E-03
hsa-miR-1246	1.86	9.06E-05	hsa-miR-149-5p	-0.71	8.60E-03
hsa-miR-223-5p	2.06	2.44E-04	hsa-miR-27b-3p	-0.31	9.28E-03
hsa-miR-505-3p	1.55	2.45E-04	hsa-miR-148b-3p	-0.43	9.50E-03
hsa-miR-142-3p	2.2	3.25E-04	hsa-miR-574-5p	-0.53	1.19E-02
hsa-miR-155-5p	1.46	7.66E-04	hsa-miR-3909	-0.59	1.19E-02
hsa-miR-3960	2.5	1.41E-03	hsa-miR-23b-3p	-0.29	1.36E-02
hsa-miR-324-5p	2.18	1.51E-02			
hsa-miR-1-3p	3.46	1.89E-02			

The complete differential expression output and filtered datasets are provided in Appendix Raw Data File 1.

### Dysregulated miRNAs enriched in neurodevelopmental and immune pathways

**Fig. 2:**
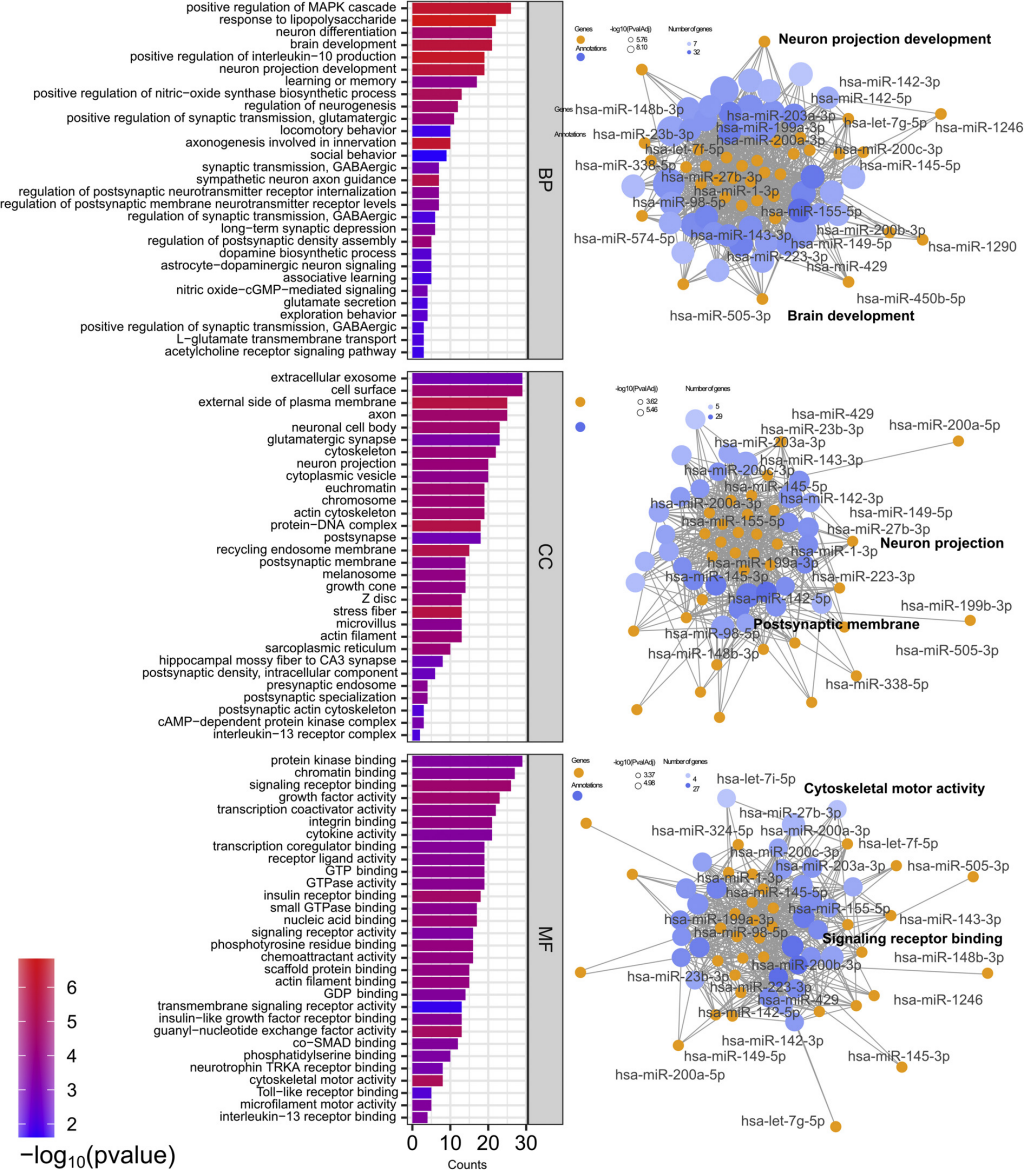
**Gene ontology enrichment analysis of differentially expressed miRNAs in ASD.** Gene ontology enrichment analysis of the ASD-differentially expressed genes in relation to biological processes (BP), cellular components (CC), and molecular functions (MF) using DAVID Bioinformatics Resources database via GeneCodis4. The top 30 enriched terms in each category are visualized in the bar graphs created in SRplot to highlight the significantly associated terms. The corresponding network graphs illustrate the relationships between significantly enriched pathway terms and their associated miRNAs.

Gene ontology (GO) analysis of target genes revealed enrichment in key neurodevelopmental processes, including axon guidance, neurogenesis, synapse organization, and glial cell differentiation (Figure 2). At the cellular level, these miRNAs were associated with components such as dendritic spines and neuronal projections, pointing to involvement in circuit connectivity. Molecular functions enriched included mRNA binding and kinase activity, relevant to neuroplasticity and intracellular signalling.

Pathway enrichment analysis using KEGG, Reactome, and WikiPathways demonstrated that dysregulated miRNAs were enriched in neurotrophin signalling, mTOR, MAPK, and TNF-alpha signalling (Supplementary Figure S3). These pathways are consistently reported as disrupted in ASD and are central to brain development, immune signalling, and metabolic regulation.

### Salivary miRNA signatures reflect systemic and neurological comorbidities

Disease enrichment analysis revealed that target genes of ASD-dysregulated miRNAs were also associated with several conditions comorbid with ASD, including epilepsy, Alzheimer's disease, inflammatory bowel disease, and autoimmune disorders (Supplementary Figure S4). For instance, miR-223-5p and miR-155-5p are upregulated in both ASD and inflammatory disorders, suggesting shared regulatory mechanisms involving immune dysregulation and neuroinflammation.

These findings suggest that salivary miRNAs reflect not only ASD-specific pathophysiology but also systemic disease susceptibility, reinforcing their role as transdiagnostic molecular indicators.

### Salivary microbial composition differs between ASD and controls

**Fig. 3:**
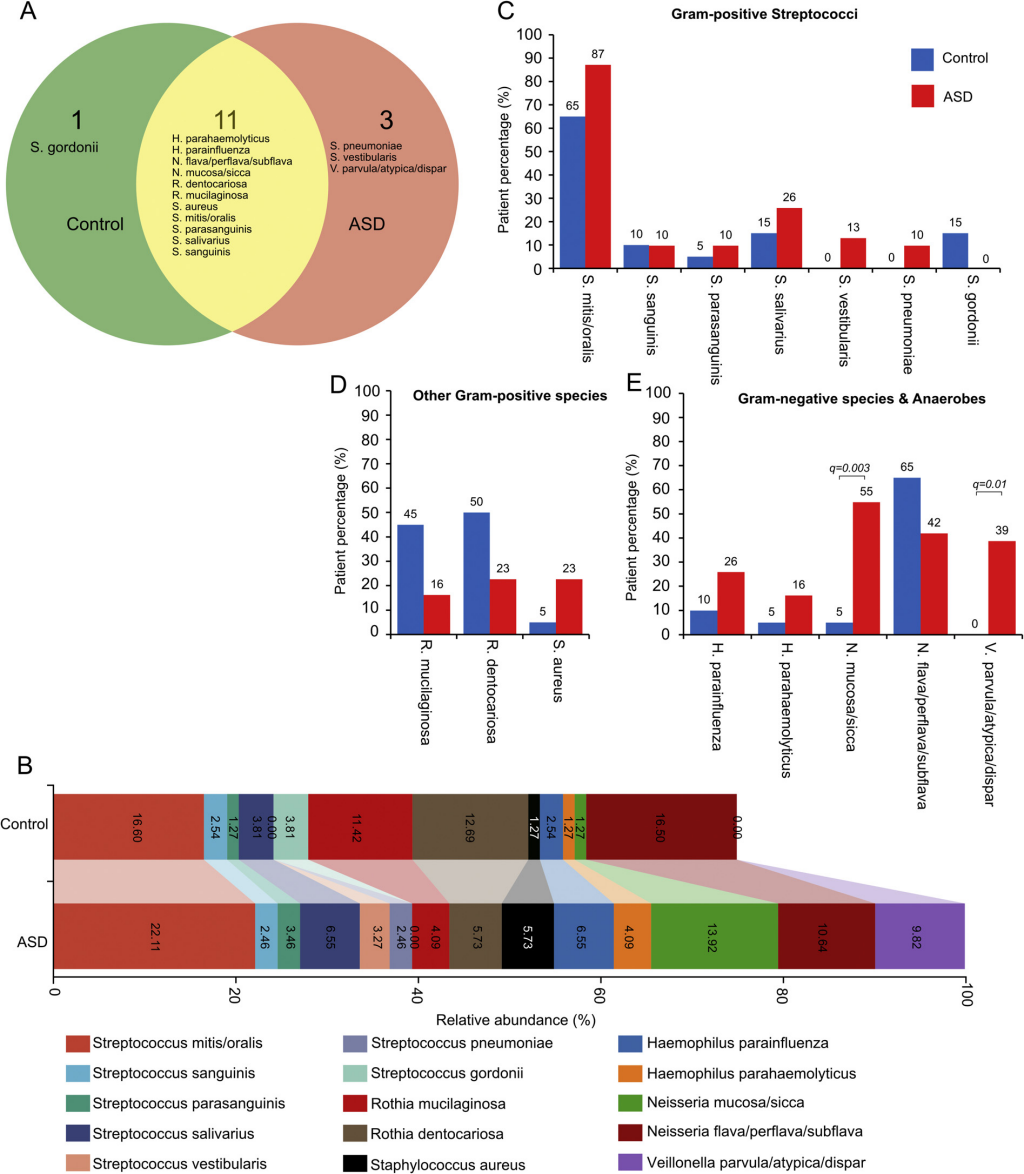
**Salivary microbial composition in ASD and control children.** (A) Venn diagram showing the number of salivary bacterial species detected exclusively in the control group, exclusively in the ASD group, and shared between both groups. (B) Stacked bar chart showing the relative abundance (%) of the predominant bacterial species detected in saliva from the control and ASD groups. Coloured bands represent individual bacterial taxa, and their widths indicate relative abundance within each group. (C) Patient percentage (%) of major Gram-positive streptococcal species detected in control and ASD samples. (D) Patient percentage (%) of other Gram-positive species detected in control and ASD samples. (E) Patient percentage (%) of Gram-negative species and anaerobes detected in control and ASD samples. Between-group differences in bacterial detection frequencies in panels C–E were evaluated using Fisher’s exact test with Benjamini–Hochberg false discovery rate (FDR) correction across species; only taxa remaining significant after FDR correction are indicated in the figure (Neisseria mucosa/sicca, q = 0.003; Veillonella parvula/atypica/dispar, q = 0.01). Part of the data visualization was conducted using SRplot, a free online platform for data visualization and graphing.

Mass spectrometry-based microbial profiling identified differences in salivary bacterial composition between ASD and control groups (Figure 3). Between-group bacterial detection frequencies were evaluated using Fisher’s exact test with Benjamini–Hochberg FDR correction across species. After correction for multiple testing, Neisseria mucosa/sicca and Veillonella parvula/atypica/dispar remained significantly more frequently detected in ASD samples (Figure 3C-E). Moreover, certain microbial taxa showed correlations with miRNA expression levels (Figure 5E, F), indicating possible host-microbe-miRNA interactions.

### ASD children show poorer oral health and greater behavioural barriers

**Fig. 4:**
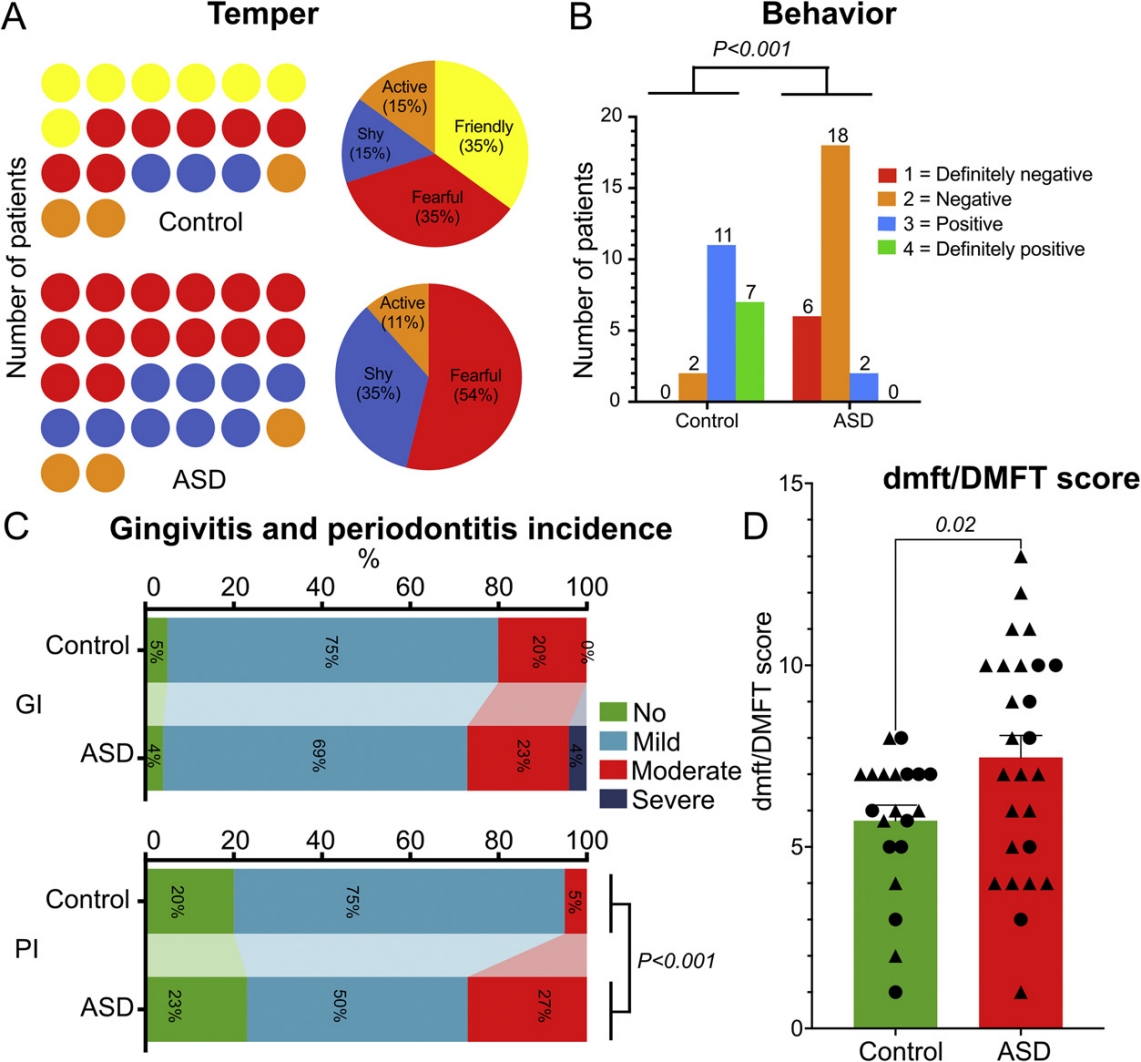
**Temper/Behavioural characteristics and oral health outcomes in ASD versus control groups**. (A) Distributions and percentages of temper scores during oral examination across the 2 study groups. (B) Bar graph showing the patient counts for the different behaviour categories across the 2 study groups. (C) Gingival index (GI) and periodontal index (PI) scores across the 2 study groups. (D) The dmft/DMFT index values across the 2 study groups, presented as mean ± SEM with individual participant data points overlaid; black circles indicate females and black triangles indicate males. Between-group comparisons were performed using Fisher’s exact test for behavioural, gingival, and periodontal categorical distributions, and an unpaired t-test with Welch’s correction for dmft/DMFT. The data visualization was conducted using SRplot, a free online platform for data visualization and graphing.

Children with ASD exhibited statistically significantly higher dmft/DMFT scores and more pronounced behavioural and periodontal challenges than controls (Figure 4). Specifically, dmft/DMFT scores differed significantly between groups (*p* = .02). Behavioural cooperation and periodontal index distributions also differed significantly between groups (*p* < .001 for both), whereas gingival index distribution did not differ significantly. These findings indicate greater behavioural barriers together with poorer oral health in the ASD group.

### miRNA expression correlates with caries burden

**Fig. 5:**
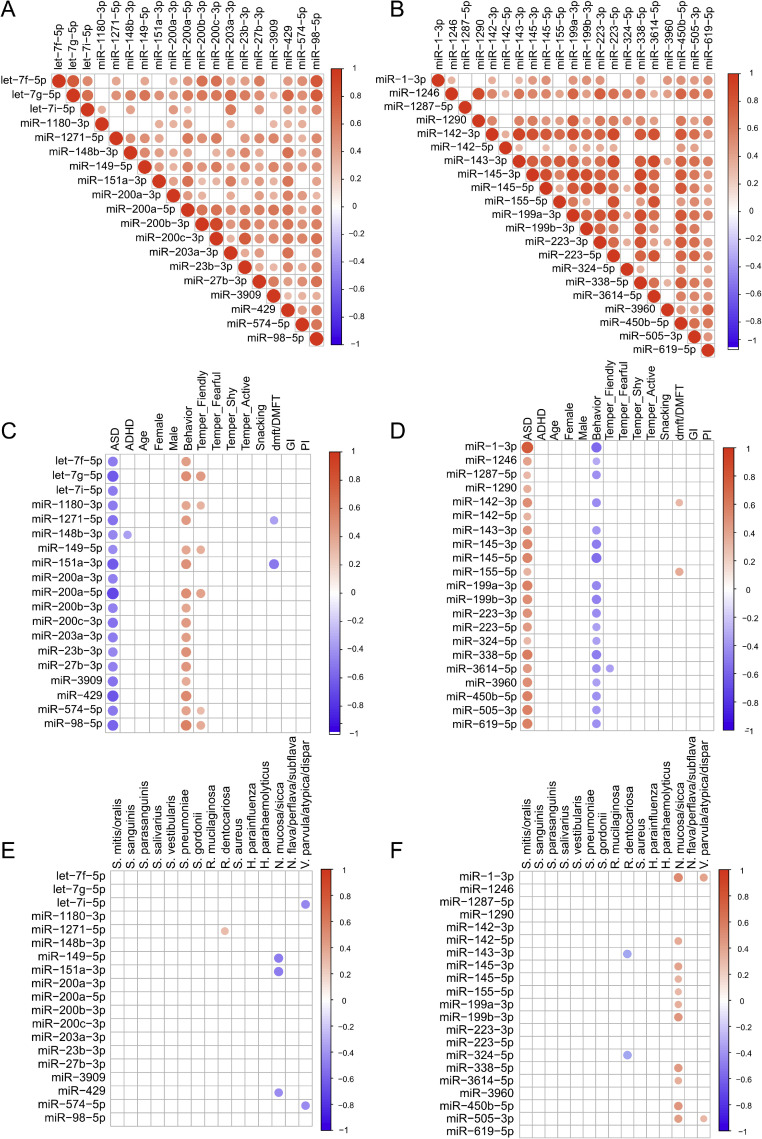
**Correlation matrix of salivary miRNAs, behavioural, and oral health variables.** The upper panel shows the heatmaps for Spearman correlations between the expression levels of the 19 downregulated (A) and 21 upregulated (B) miRNAs in ASD children. The middle panel shows heatmaps of Spearman correlations between the demographic/behavioural/oral health variables and the expression levels of the 19 downregulated (C) and 21 upregulated (D) miRNAs in ASD children. The lower panel shows heatmaps of Spearman correlations between the bacterial species detected and the expression levels of the 19 downregulated (E) and 21 upregulated (F) miRNAs in ASD children. FDR-significant correlations (Benjamini–Hochberg-adjusted q < 0.05) are indicated by circles, with larger circles representing stronger statistical support. Correlation coefficients are represented by the colour bar and the corresponding colour intensity of the circles. Data visualization was conducted using SRplot, a free online platform for data visualization and graphing.

Spearman correlation analyses were performed across the 6 predefined Figure 5 families, and p-values were adjusted within each family using the Benjamini–Hochberg FDR method. After FDR correction within the clinical correlation families, miR-151a-3p and miR-1271-5p remained inversely associated with dmft/DMFT, whereas miR-155-5p and miR-142-3p remained positively associated with dmft/DMFT. In follow-up exploratory multiple linear regression using the pooled cohort (Table 2), miR-151a-3p and behaviour remained significant predictors of dental caries burden, while the overall model remained statistically significant (adjusted R^2^ = 0.31, *P* = .003).

**Table 2:** Exploratory multiple linear regression analysis of variables predicting dental caries burden (dmft/DMFT)

Predictor	Unstandardized B	Standardized β	95% CI for B	*P*-value	Adjusted R^2^
miR-142-3p	-0.013	-0.107	-0.064 to 0.037	.594	0.31
miR-155-5p	0.007	0.182	-0.008 to 0.022	.331	
miR-1271-5p	-0.006	-0.147	-0.018 to 0.007	.35	
**miR-151a-3p**	**-0.008**	**-0.408**	**-0.016 to -0.001**	**.029**	
ASD	-2.301	-0.418	-4.944 to 0.342	.086	
**Behaviour**	**-1.489**	**-0.492**	**-2.810 to -0.168**	**.028**	
PI	0.413	0.094	-0.863 to 1.689	.516	

Unstandardized regression coefficients (B), standardized beta coefficients (β), 95% confidence intervals (CI) for B, *p*-values, and adjusted R^2^ are shown. Variables were selected based on dmft/DMFT correlation screening (miRNAs) and clinical relevance. The overall model was statistically significant (*p* = .003). As this model was used as a follow-up exploratory analysis on a reduced candidate set, no additional multiplicity correction was applied. Statistically significant predictors are shown in bold.

## Discussion

In this study, we identified a distinct salivary microRNA (miRNA) signature that differentiates children with autism spectrum disorder (ASD) from neurotypical peers. By integrating miRNA profiling, gene enrichment analyses, salivary microbiome data, and oral health indices, we reveal a multi-layered molecular and phenotypic network that may contribute to ASD-related biological features and associated oral health vulnerability.

### Principal findings of the present study

Among the dysregulated salivary miRNAs identified in this study, miR-151a-3p emerged as an important informative signal associated with oral health burden in children with ASD. It was significantly downregulated in ASD, remained inversely associated with dmft/DMFT after correction of the clinical-correlation analyses, and remained significant in the regression model, together with behaviour. These findings are consistent with the interpretation that the higher caries burden observed in children with ASD may be related not only to diagnostic status, but also to associated behavioural and molecular features. Moreover, miR-151a-3p showed strong positive correlations with several other downregulated miRNAs, particularly miR-200a-5p, miR-203a-3p, miR-429, miR-149-5p, and let-7g-5p. This pattern is biologically informative because members of the miR-200 family, including miR-200a and miR-429, have documented roles in dental development, oral health, epithelial regulation, and barrier-related pathways,^26^, ^27^, ^28^, ^29^ whereas miR-203a and miR-149-5p convey major regulatory roles related to oral infections and inflammatory conditions and cytokine responses.^30^, ^31^, ^32^ This network-level pattern also fits with the biology of miR-151-3p itself, which has been shown to target STAT3 and suppress LPS-induced IL-6 cytokine production.^33^ Although these mechanistic links were not directly tested here, the coordinated downregulation of miR-151a-3p together with these functionally related miRNAs is consistent with the possibility that it forms part of a broader dysregulated inflammatory and barrier-related milieu associated with oral-health vulnerability in ASD. Prior saliva-based studies have also identified the relevance of miR-151a-3p to neurodevelopmental disorders,^10^^,^^34^ further supporting the biological plausibility of our findings and highlighting miR-151a-3p as a compelling salivary marker in ASD.

### Comparison with previous saliva-based ASD studies

Importantly, several of the dysregulated miRNAs identified in our prioritized salivary signature overlap with previously reported saliva-based ASD findings, thereby providing independent confirmation in our cohort. These include miR-151a-3p, as well as multiple miRNAs reported in independent salivary profiling studies, such as miR-1246, miR-199a-3p, miR-199b-3p, miR-223-5p, miR-143-3p, miR-142-3p, miR-145-5p, miR-142-5p, miR-155-5p, miR-200b-3p, miR-203a-3p, and miR-149-5p.^10^^,^^35^, ^36^, ^37^ Taken together, these overlaps strengthen the biological relevance of saliva as a non-invasive and informative biofluid for ASD biomarker discovery. Notably, our study adds an important layer of interpretation by showing that these salivary miRNAs are not only dysregulated, but also prioritized as putative regulators of genes with strong ASD relevance through integrated miRDB/SFARI analysis. To our knowledge, no single dedicated curated resource currently links saliva-based ASD miRNAs directly to ASD-gene prioritization in this way, making this combined framework a useful advance for interpreting the potential functional relevance of salivary miRNA biomarkers in ASD.

### Comparison with previous non-salivary ASD studies

Several salivary microRNAs (miRNAs) dysregulated in our study, such as miR-155-5p, miR-142-3p, and miR-223-5p, have also been reported in non-salivary samples from patients with ASD and other neuroinflammatory conditions. For instance, miR-155-5p is upregulated in the amygdala of children with ASD, suggesting its role in neuroinflammation.^38^ Similarly, miR-142-3p was reported to regulate T cell differentiation and inflammation in autoimmune neuroinflammation.^39^ Additionally, miR-223-5p is elevated in the blood of ASD patients, indicating its involvement in systemic immune responses.^40^ These findings from blood, brain, and immune cell studies reinforce the systemic relevance of these miRNAs in ASD. Notably, miR-1271-5p, which we identified as significantly downregulated in ASD saliva, has also been found down-regulated in the lymphocytes and blood of schizophrenia patients,^41^^,^^42^ and it has been implicated in neuronal differentiation, synaptic connectivity, and neurotransmission.^43^ Although previously studied in neuronal cells and in serum, this is the first report to identify miR-1271-5p dysregulation in ASD saliva, potentially expanding its clinical utility as a non-invasive neurodevelopmental biomarker.

### Host–microbial associations in ASD

At the host–microbe interface, our salivary microbial data add a more specific oral ecological dimension to the miRNA findings. After appropriate statistical correction across species, only 2 taxa remained significantly more frequent in ASD samples: Neisseria mucosa/sicca and Veillonella parvula/atypica/dispar. This is noteworthy because these taxa occupy distinct ecological niches in the oral cavity. Oral Neisseria species are well-recognized nitrate-reducing commensals that contribute to nitrate–nitrite–nitric oxide metabolism and have been linked to oral ecological homeostasis and health-associated biofilm function, whereas Veillonella species are lactate-utilizing biofilm organisms that have repeatedly been associated with cariogenic and dysbiotic oral communities.^44^, ^45^, ^46^, ^47^ Rather than indicating a simple shift toward “pathogenic” bacteria, our data are more consistent with a broader reorganization of the salivary ecosystem in ASD, involving taxa linked to altered biofilm metabolism, host interactions, and oral niche adaptation. These observations should nevertheless be interpreted cautiously, as this was an observational cross-sectional study and the present data do not establish whether the microbial shifts contribute directly to oral disease, reflect altered host biology, or both.

These findings partly parallel earlier saliva-based ASD microbiome work. Ragusa and colleagues reported altered salivary microbiome structure in autistic children, including reduced Tannerella abundance and a negative association with miR-141-3p.^48^ In our cohort, Tannerella was not detected, whereas the most robust statistically supported signals involved Neisseria mucosa/sicca and Veillonella parvula/atypica/dispar. Importantly, the corrected bacteria–miRNA correlation analysis suggested that these species were linked to miRNAs with documented roles in host immune and microbial-response pathways. For Neisseria mucosa/sicca, the strongest biologically interpretable correlations involved miR-142-5p and miR-149-5p. Notably, miR-142-5p is involved in immune-cell and inflammatory regulation, whereas miR-149-5p can dampen TLR/MyD88/NF-κB-driven inflammatory signaling.^49^, ^50^, ^51^, ^52^ For Veillonella parvula/atypica/dispar, the strongest biologically interpretable correlations involved let-7i-5p and miR-574-5p; let-7i has been linked to TLR4-related epithelial responses during microbial challenge, and miR-574-5p has been implicated in infection-related inflammatory signaling.^53^, ^54^, ^55^, ^56^ Taken together, these patterns are consistent with the possibility that altered salivary miRNA regulation and shifts in oral microbial ecology may coexist within a shared host–microbe response framework in ASD.

### Broader biological and translational implications

Overall, we identified 125 differentially expressed salivary miRNAs in ASD, several of which are known regulators of neurodevelopment, immune signalling, and mucosal integrity. Gene ontology and disease enrichment analyses confirmed that ASD-associated miRNAs target networks involved in neurogenesis, axonal guidance, synaptic signalling, and intracellular pathways. These findings align with existing genomic data implicating convergent neurodevelopmental disruptions in ASD.^7^^,^^57^ Furthermore, disease ontology terms enriched among target genes included epilepsy, IBD, and neurodegeneration, frequent comorbidities in ASD.

Our findings also highlight intriguing overlaps between salivary miRNA dysregulation in ASD and miRNAs previously shown to be influenced by parental environmental exposures. Recent evidence from a pregnancy cohort indicates that maternal lifetime stressors are associated with altered expression of breast milk-derived miRNAs, including upregulation of miR-155-5p.^58^ Consistently, we identified miR-155-5p as significantly upregulated in the saliva of ASD children. Given the emerging role of milk miRNAs in modulating immune and neurodevelopmental pathways,^59^^,^^60^ these findings raise the possibility that early-life exposure to stress-responsive miRNAs may contribute to ASD-related molecular programming.

Moreover, several miRNAs we identified in ASD saliva samples, including miR-19a-3p, miR-3613-3p, miR-150-5p, miR-126-3p, and miR-499a-5p, overlap with those found dysregulated in the sperm of fathers of children with ASD.^61^ More recent mechanistic studies in the mouse model have demonstrated that early-life trauma can alter sperm RNA profiles, leading to behavioural and metabolic changes in offspring through epigenetic inheritance.^62^ This convergence is consistent with the emerging model that paternal miRNAs can influence offspring neurodevelopment, and salivary miRNA profiles in ASD may reflect a molecular echo of inherited regulatory disruptions.

### Study limitations and future directions

This study presents a comprehensive investigation of salivary miRNAs in ASD, integrating molecular, microbial, and clinical dental data. However, the study is limited by a moderate sample size, which restricts stratified analyses by ASD severity or comorbidity. While gene and disease enrichments provide mechanistic insights, experimental validation of miRNA-target interactions remains necessary. Microbial data were limited to species identification, and functional metagenomics could further refine our understanding of host-microbe-miRNA interactions in children with ASD.

## Conclusion

This study identifies a robust panel of differentially expressed salivary miRNAs in children with autism spectrum disorder, including both previously reported and newly prioritized ASD-relevant candidates. We further show that selected salivary miRNAs are linked to oral health burden, particularly dental caries, and may reflect interactions among neurodevelopmental, immune, and oral ecological factors in ASD. These findings support the use of saliva as a non-invasive window into ASD-associated biology, with potential applications in biomarker discovery and risk stratification. Future longitudinal studies are warranted to clarify the predictive value, developmental trajectories, and functional significance of these salivary miRNAs in ASD.

## Ethical approval

This human observational study conforms to the STROBE guidelines. The study was conducted in accordance with the principles of the Declaration of Helsinki and approved by the Institutional Review Board (IRB) at Imam Abdulrahman Bin Faisal University, Saudi Arabia (IRB-2021-02-463). Written informed consent was obtained from the children’s legal guardians.

## Funding

Deputyship for Research and Innovation, the Ministry of Education in Saudi Arabia, through project number IF-2020-014-Dent at Imam Abdulrahman bin Faisal University/College of Dentistry. The funding body had no role in the study design, data collection, analysis and interpretation, report writing, or the decision to submit the article for publication.

## Data availability

All data supporting the findings of this study are included in the main manuscript and its Supplementary Information file. The raw datasets underlying the analyses are available from the corresponding authors upon reasonable request. No custom computer code or algorithm central to the conclusions of this study was used.

## Author contributions

Omar Omar: Conceptualization, Data curation, Formal analysis, Funding acquisition, Investigation, Methodology, Project administration, Visualization, Writing – original draft, Writing – review and editing. Reem Yussuf AlJindan: Conceptualization, Resources, Formal analysis, Methodology, Supervision, Writing – review and editing. Sumit Rajinder: Conceptualization, Funding acquisition, Formal analysis, Investigation, Methodology, Writing – review and editing. Balu Kamaraj: Conceptualization, Formal analysis, Data curation, Software, Writing – review and editing. Jehan AlHumaid: Conceptualization, Funding acquisition, Investigation, Methodology, Supervision, Writing – review and editing.

## Conflict of interest

None disclosed.

## Appendix Supplementary materials

Image, application 1

Image, application 2

Image, application 3

Image, application 4

Image, application 5

Image, application 6

Image, application 7

Image, application 8

Image, application 9

## Footnotes

Supplementary material associated with this article can be found in the online version at doi:10.1016/j.identj.2026.109632.
