# Supplementary material for: Salivary MicroRNA Reflects Neurodevelopment and Oral Health Traits in Children With Autism
Source: Int Dent J. 2026 May 20;76(4):109632. doi: 10.1016/j.identj.2026.109632 (PMC13217594; doi:10.1016/j.identj.2026.109632)
Supplement: Supplementary file 9 [file mmc9.docx]

**Supplementary Information**

**Salivary microRNA reflects neurodevelopment and oral health traits in children with autism**

Omar Omar,^a,*^ Reem Yussuf AlJindan,^b^ Sumit Rajinder,^c^ Balu Kamaraj,^d^ Jehan AlHumaid,^c,#^

^a^Department of Biomedical Dental Science, College of Dentistry, Imam Abdulrahman Bin Faisal University, Dammam, Saudi Arabia

^b^Department of Microbiology, College of Medicine, Imam Abdulrahman Bin Faisal University, Dammam, Saudi Arabia

^c^Department of Preventive Dental Science, College of Dentistry, Imam Abdulrahman Bin Faisal University, Dammam, Saudi Arabia

^d^Department of Dental Education, College of Dentistry, Imam Abdulrahman Bin Faisal University, Dammam, Saudi Arabia

**Running Title:** Salivary miRNAs and oral health in autism

^*^**Corresponding Author 1:** Omar Omar, BDS, PhD, Associate Professor

Department of Biomedical Dental Sciences, College of Dentistry

Imam Abdulrahman Bin Faisal University. P.O. Box 1982, Dammam-31441, Saudi Arabia.

Phone: +966 58 144 0342; Email: [omomar@iau.edu.sa](mailto:omomar@iau.edu.sa); ORCID: 0000-0002-2610-1294

^#^**Corresponding Author 2:** Jehan AlHumaid, BDS, M.A.Ed, DScD, Professor

Department of Preventive Dental Science, College of Dentistry

Imam Abdulrahman Bin Faisal University. P.O. Box 1982, Dammam-31441, Saudi Arabia.

Phone: +966 50 584 9857; Email: [jaalhumaid@iau.edu.sa](mailto:jaalhumaid@iau.edu.sa); ORCID: 0000-0002-8263-6285

**Supplementary Figures**

**
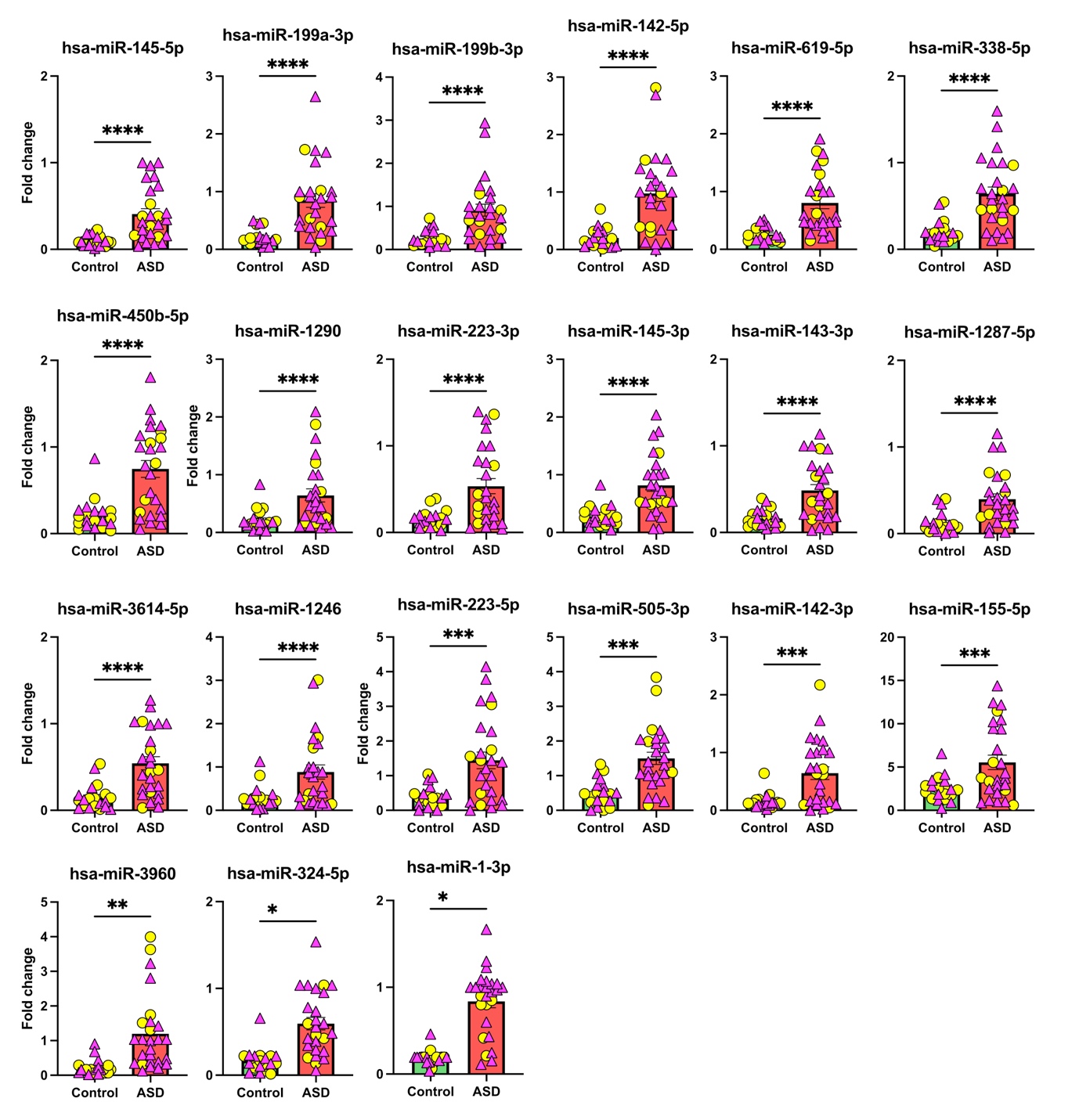
**

**Supplementary Figure S1. Upregulated salivary microRNAs in children with ASD.** Bar graph illustrating normalized expression levels (log2-transformed) of miRNAs significantly upregulated in ASD compared to controls (FDR < 0.05). Data are shown as mean ± SEM with individual participant data points overlaid; black circles indicate females and black triangles indicate males; *n* = 26 ASD, 20 controls. Statistical analysis was performed using DESeq2 with multiple testing correction.

**
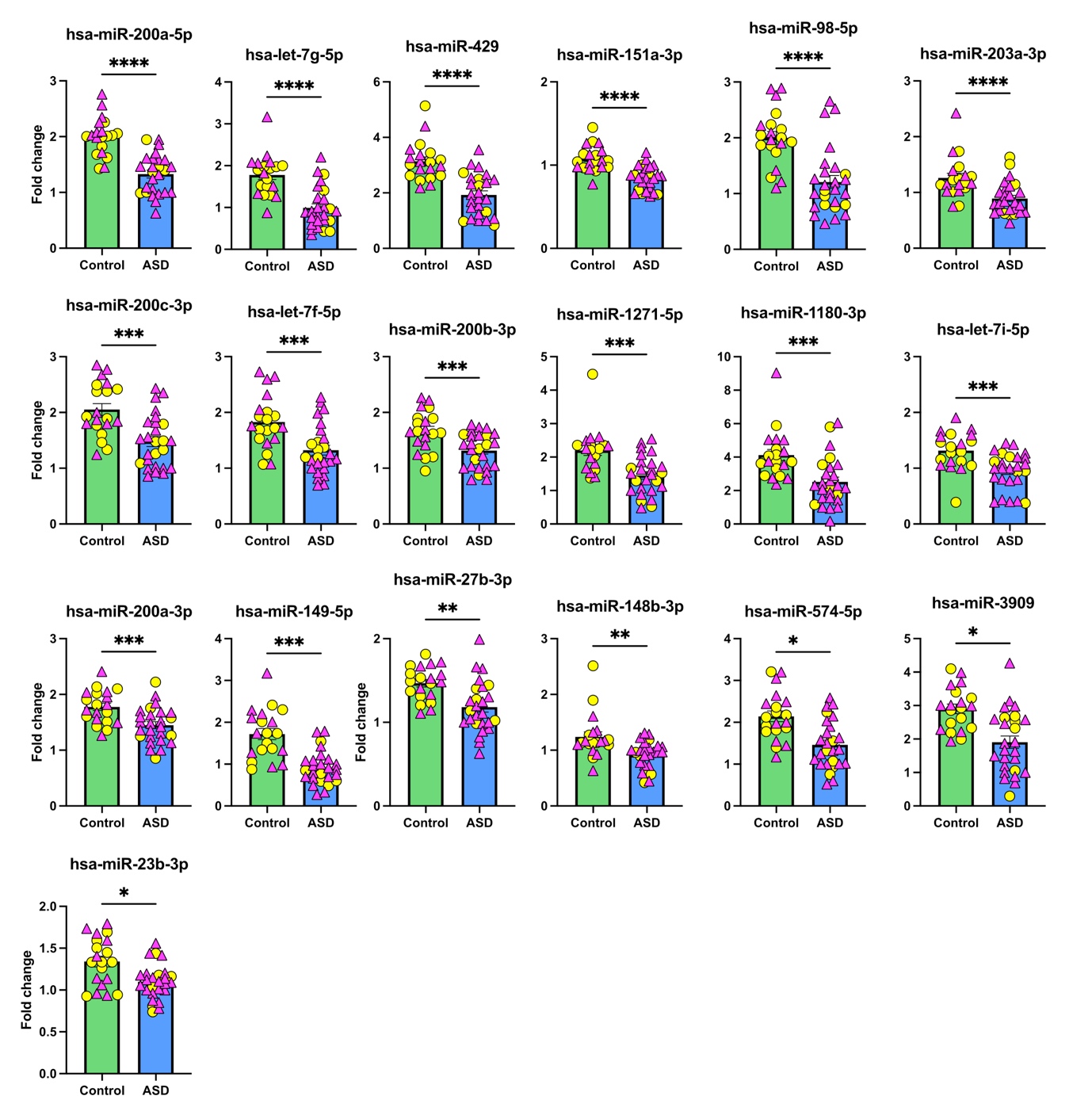
**

**Supplementary Figure S2. Downregulated salivary microRNAs in children with ASD.** Bar graph showing normalized expression levels (log2-transformed) of miRNAs significantly downregulated in ASD subjects compared to controls (FDR < 0.05). Data are presented as mean ± SEM with individual participant data points overlaid; black circles indicate females and black triangles indicate males; *n* = 26 ASD, 20 controls. Statistical significance determined using DESeq2 with Benjamini-Hochberg correction.


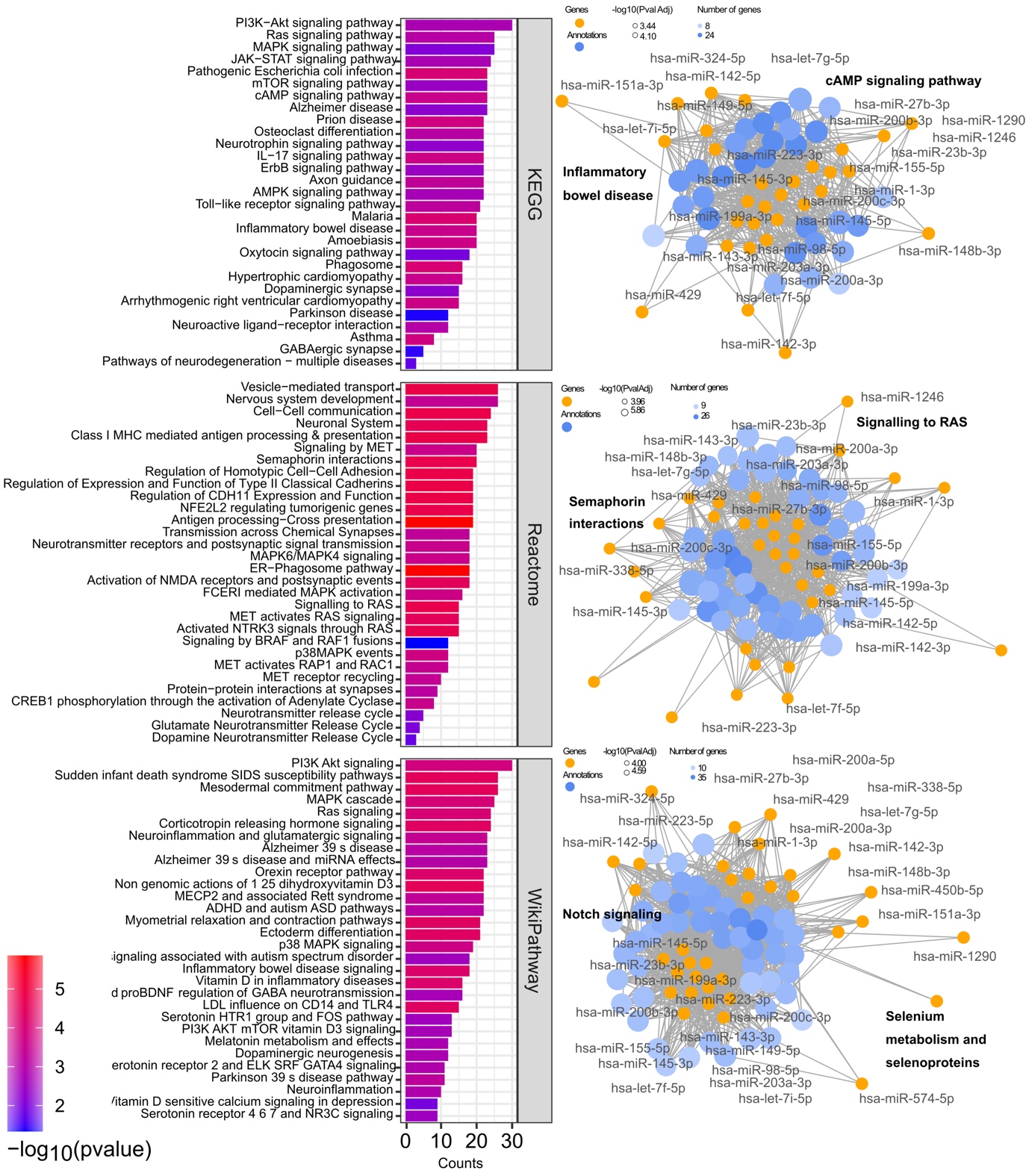


**Supplementary Figure S3. Pathway enrichment analysis of differentially expressed miRNAs in ASD.** Pathway enrichment analysis of the ASD-differentially expressed genes using KEGG, Reactome, and WikiPathways databases via GeneCodis4. The top 30 enriched terms in each category are visualized in the bar graphs created in SRplot to highlight significantly associated terms. The corresponding network graphs illustrate the relationships between significantly enriched pathway terms and their associated miRNAs.

**
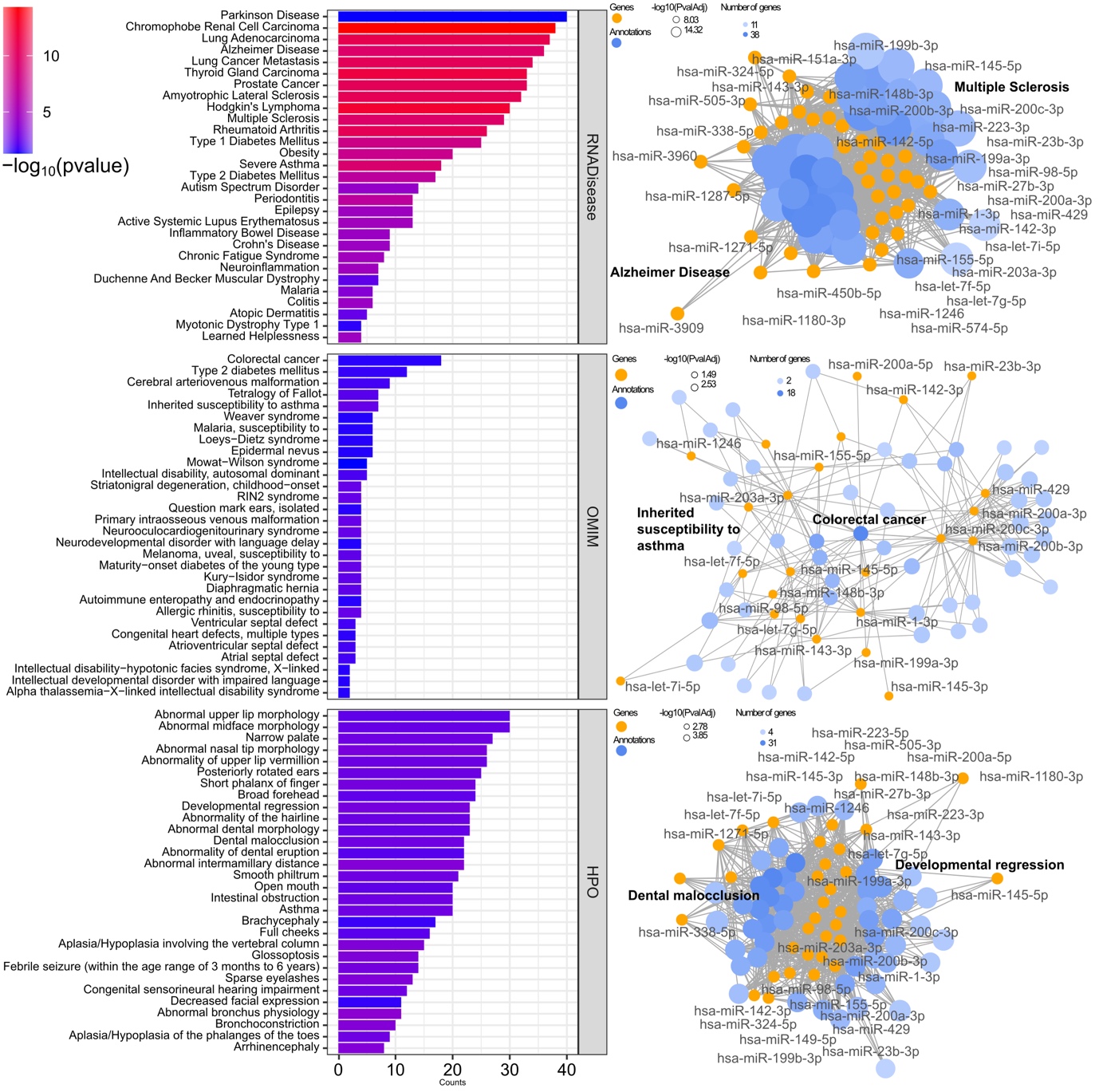
**

**Supplementary Figure S4. Disease and phenotype enrichment analysis of differentially expressed miRNAs in ASD.** Disease and phenotype enrichment analysis of differentially expressed genes using RNADisease, Online Mendelian Inheritance in Man (OMIM), and Human Phenotype Ontology (HPO) databases via GeneCodis4. The top 30 enriched terms in each category are visualized in the bar graphs created in SRplot to highlight significantly associated terms. The corresponding network graphs illustrate the relationships between significantly enriched pathway terms and their associated miRNAs.

**Supplementary raw research data files descriptions**

**Appendix 1 - Raw Data File 1_NGS output and DEGs miRDB-SFARI-filtered**

This Excel file presents the complete workflow of the miRNA sequencing analysis comparing saliva samples from ASD and control subjects. The first sheet, “ALL DETECTED (2229 MiR)”, lists all 2229 miRNAs detected in the samples. From these, miRNAs with missing adjusted p-values (NA) were removed, resulting in 646 miRNAs retained in the second sheet, “ALL_REMOVED NA (646 MiR)”. Among these, miRNAs showing a statistically significant difference between ASD and control groups were selected, resulting in 125 differentially expressed miRNAs compiled in “Sig. Up & DownReg (125 MiR)”. These significant miRNAs were then subdivided into those upregulated in ASD “Sig. UpReg (97 MiR)” and those downregulated in ASD “Sig. DownReg (28 MiR)” compared to controls. To further prioritize biologically relevant candidates, the upregulated and downregulated miRNAs were separately filtered based on functional annotation using the miRDB and SFARI databases. Specifically, upregulated miRNAs with >90% target prediction scores in miRDB and whose predicted targets are verified ASD-related genes in SFARI (21 miRNAs) were compiled in “Sig. UpReg miRDB-SFARI (21 MiR)”, while the downregulated miRNAs meeting the same criteria (19 miRNAs) were compiled in “Sig. DownReg miRDB-SFARI (19 MiR)”. This structured approach enables the identification of miRNAs with both statistical significance and potential functional relevance to ASD.

**Appendix 2 - Raw Data File 2_40 MiRNAs Enrich-Output-GO**

This Excel file contains the complete Gene Ontology (GO) enrichment analysis of ASD-associated differentially expressed miRNAs, performed using the GeneCodis4 webtool. Full enrichment results for the significantly (P<0.05) enriched Biological Processes, Cellular Components, and Molecular Functions are provided in the sheets titled “SIG-Enrich-Output-GO_BP”, “SIG-Enrich-Output-GO_CC”, and “SIG-Enrich-Output-GO_MF”, respectively. The sheets “TOP-SELECTED-30-GO-BP”, “TOP-SELECTED-30-GO-CC”, and “TOP-SELECTED-30-GO-MF” summarize the top 30 enriched terms in each category, selected based on statistical significance level and biological relevance.

**Appendix 3 - Raw Data File 3_40 MiRNAs Enrich-Output-PATHWAYS**

This Excel file contains the pathway enrichment analysis of ASD-associated differentially expressed miRNAs, performed using the GeneCodis4 webtool. Full enrichment results for the significantly (P<0.05) enriched pathways based on KEGG, Reactome, and WikiPathways databases are provided in the sheets titled “SIG-Enrich-Output-KEGG)”, “SIG-Enrich-Output-Reactome”, and “SIG-Enrich-Output-WikiPathways”, respectively. The sheets “TOP-SELECTED-30-KEGG”, “TOP-SELECTED-30-Reactome”, and “TOP-SELECTED-30-WikiPathway” summarize the top 30 enriched terms in each pathway database, selected based on statistical significance and biological relevance.

**Appendix 4 - Raw Data File 4_40 MiRNAs Enrich-Output-RNADisease OMIM HPO**

This Excel file contains disease and phenotype enrichment analysis of ASD-associated differentially expressed miRNAs, performed using the GeneCodis4 webtool. Full enrichment results for the significantly (P<0.05) enriched diseases and phenotypes based on RNADisease, OMIM, and HPO databases are provided in the sheets titled “SIG-Enrich-Output-RNADisease)”, “SIG-Enrich-Output-OMIM”, and “SIG-Enrich-Output-HPO”, respectively. The sheets “TOP-SELECTED-30- RNADisease”, “TOP-SELECTED-30-OMIM”, and “TOP-SELECTED-30-HPO” summarize the top 30 enriched terms in each database, selected based on statistical significance and biological relevance.

**Appendix 5 - Raw Data File 5_Clinical and Bacterial Data**

This Excel file presents the full raw data of the analyzed clinical parameters and bacterial species in the saliva samples from ASD and control subjects.

**Appendix 6 - Raw Data File 6_Correlation Input Data**

This Excel file contains the raw data used for correlation analyses involving salivary microRNAs (miRNAs) in children with autism spectrum disorder (ASD) and control subjects. The dataset comprises six sheets, each corresponding to a specific set of analyses. The first sheet evaluates pairwise correlations among ASD-upregulated miRNAs. The second sheet evaluates correlations among ASD-downregulated miRNAs. The third and fourth sheets assess correlations between the expression of upregulated and downregulated miRNAs, respectively, and clinical parameters such as caries index, gingival and plaque indices, cognitive and autism-related behavioral scores. The fifth sheet for upregulated miRNAs alongside detected bacterial taxa, allowing for correlation analysis between miRNA expression and microbial abundance. Similarly, the sixth sheet provides the corresponding data for downregulated miRNAs.

**Appendix 7 - Raw Data File 7_Correlation Output Data_Corrected**

This Excel file contains the complete output of the corrected Spearman correlation analyses performed for Figure 5. The file comprises six sheets corresponding to the six predefined correlation families: pairwise correlations among downregulated miRNAs (Fig5A_full data), pairwise correlations among upregulated miRNAs (Fig5B_full data), correlations between downregulated miRNAs and clinical variables (Fig5C_full data), correlations between upregulated miRNAs and clinical variables (Fig5D_full data), correlations between downregulated miRNAs and bacterial taxa (Fig5E_full data), and correlations between upregulated miRNAs and bacterial taxa (Fig5F_full data). Each sheet reports the paired variables, Spearman’s rho, nominal unadjusted p-value, Benjamini–Hochberg FDR-adjusted q-value, and whether the correlation remained significant after FDR correction. The Summary sheet provides an overview of the total number of tested correlations and the number of FDR-significant associations within each Figure 5 panel.
